# Exploring Beneficial Properties of Haskap Berry Leaf Compounds for Gut Health Enhancement

**DOI:** 10.3390/antiox13030357

**Published:** 2024-03-17

**Authors:** Szymon Sip, Anna Sip, Piotr Szulc, Marek Selwet, Marcin Żarowski, Bogusław Czerny, Judyta Cielecka-Piontek

**Affiliations:** 1 Department of Pharmacognosy and Biomaterials, Faculty of Pharmacy, Poznań University of Medical Sciences, Rokietnicka 3, 60-806 Poznań, Poland; szymonsip@ump.edu.pl; 2 Department of Biotechnology and Food Microbiology, Poznan University of Life Sciences, Wojska Polskiego 48, 60-627 Poznań, Poland; anna.sip@up.poznan.pl; 3 Department of Agronomy, Poznań University of Life Sciences, Dojazd 11, 60-632 Poznań, Poland; piotr.szulc@up.poznan.pl; 4 Department of Soil Science and Microbiology, Poznań University of Life Sciences, Szydłowska 50, 60-656 Poznań, Poland; marek.selwet@up.poznan.pl; 5 Department of Developmental Neurology, Poznan University of Medical Sciences, Przybyszewski 49, 60-355 Poznan, Poland; zarowski@ump.edu.pl; 6 Department of General Pharmacology and Pharmacoeconomics, Pomeranian Medical University in Szczecin, Żołnierska 48, 70-204 Szczecin, Poland; bczerny@wp.pl; 7 Department of Pharmacology and Phytochemistry, Institute of Natural Fibres and Medicinal Plants, Wojska Polskiego 71b, 60-630 Poznan, Poland

**Keywords:** haskap berry, prebiotic potential, antioxidants, by-products, delivery systems, dextran

## Abstract

This study investigates the potential of formulated systems utilising haskap berry leaf extracts and dextran as carriers, to modulate both antioxidant and enzymatic inhibitory activities and their impact on the growth of specific bacterial strains. The analysis of antioxidant capacity, assessed through ABTS, CUPRAC, DPPH, and FRAP assays, revealed varying but consistently high levels across extracts, with Extract 3 (loganic acid: 2.974 mg/g, chlorogenic acid: 1.125 mg/g, caffeic acid: 0.083 mg/g, rutin: 1.137 mg/g, and quercetin: 1.501 mg/g) exhibiting the highest values (ABTS: 0.2447 mg/mL, CUPRAC: 0.3121 mg/mL, DPPH: 0.21001 mg/mL, and FRAP: 0.3411 mg/mL). Subsequent enzymatic inhibition assays demonstrated a notable inhibitory potential against α-glucosidase (1.4915 mg/mL, expressed as acarbose equivalent), hyaluronidase (0.2982 mg/mL, expressed as quercetin equivalent), and lipase (5.8715 µg/mL, expressed as orlistat equivalent). Further system development involved integration with dextran, showcasing their preserved bioactive compound content and emphasising their stability and potential bioactivity. Evaluation of the dextran systems’ impact on bacterial growth revealed a significant proliferation of beneficial strains, particularly the *Bifidobacterium* and lactobacilli genus (Bifidobacterium longum: 9.54 × 10^7^ to 1.57 × 10^10^ CFU/mL and *Ligilactobacillus salivarius*: 1.36 × 10^9^ to 1.62 × 10^10^ CFU/mL), suggesting their potential to modulate gut microbiota. These findings offer a foundation for exploring the therapeutic applications of haskap berry-based dextran systems in managing conditions like diabetes, emphasising the interconnected roles of antioxidant-rich botanical extracts and dextran formulations in promoting overall metabolic health.

## 1. Introduction

Recent attention has turned towards the gut microbiota’s pivotal role in maintaining overall health, particularly in conditions like diabetes [1,2,3]. A thriving community of beneficial microorganisms within the gastrointestinal tract has been associated with diverse health benefits, including enhanced digestion, immune system support, and modulation of systemic inflammation [4,5]. In response, strategies to cultivate a healthy gut microbiota have become a focal point in nutrition and biomedical research, with a particular emphasis on natural compounds and dietary interventions [6,7]. In this context, prebiotics, non-digestible food components selectively stimulating the growth of beneficial microorganisms in the gut, have gained prominence for their potential to enhance gut health. Botanical sources rich in bioactive compounds, such as haskap berry leaves (*Lonicera caerulea var. kamtschatica*), are promising candidates for developing systems with the potential to modulate gut microbiome [8].

The pivotal preliminary study identified the most active variety of haskap berry leaves, showcasing high antioxidant potential and significant inhibitory activity against α-glucosidase, hyaluronidase, and lipase. Recognising the potential relevance of these findings in diabetes, our study builds upon this foundation to explore the application of haskap berry leaves in addressing aspects of this metabolic condition [9].

Haskap berry leaves present an extraordinary phytochemical profile featuring a rich array of polyphenolic compounds, including quercetin, rutin, catechins, kaempferol, and myricetin. These bioactive constituents exhibit a spectrum of potent activities, each holding profound potential in diabetes management [10,11]. Quercetin, abundantly present in haskap berry leaves, emerges as a pivotal bioactive compound with robust antioxidant properties. The observed antioxidant effects of quercetin play a crucial role in mitigating oxidative stress, a critical factor in the progression of diabetes. By scavenging free radicals and reducing reactive oxygen species, quercetin contributes to the overall reduction in oxidative stress, potentially offering protective effects against diabetes-related complications [12].

Moreover, the anti-inflammatory effects attributed to quercetin further enhance its potential therapeutic benefits in diabetes management. Chronic inflammation is intricately linked to the pathogenesis of diabetes and its complications. Quercetin’s ability to modulate inflammatory pathways may contribute to ameliorating the inflammatory processes implicated in diabetes, providing an additional layer of protection against the development and progression of diabetes-related complications [13]. The multifaceted properties of quercetin position it as a promising bioactive compound with the potential to address multiple facets of diabetes pathology, making it a valuable component of haskap berry leaf extracts for potential therapeutic applications in diabetes management. By complementing the antioxidant repertoire [14,15], rutin, a prominent bioactive compound found abundantly in haskap berry leaves, exhibits noteworthy anti-inflammatory effects contributing to its potential role in addressing diabetes progression and associated cardiovascular complications. The multifaceted properties of rutin make it a promising candidate for therapeutic applications in managing diabetes-related conditions [16,17]. Catechins, contributing to metabolic health, have been linked to improved metabolic parameters and modulation of the gut microbiota composition, influencing factors like insulin sensitivity and glucose metabolism [18,19]. Studies have suggested that catechins can positively impact metabolic health by improving insulin sensitivity. Enhanced insulin sensitivity is particularly relevant in diabetes, where insulin resistance is a central feature. Catechins may facilitate glucose uptake by tissues at the cellular level, thereby contributing to an improved overall insulin sensitivity [20]. The influence of catechins on the gut microbiota composition adds another layer of complexity to their metabolic effects. The gut microbiota plays a crucial role in metabolic processes, and alterations in its composition can impact insulin sensitivity and glucose metabolism. Catechins’ ability to modulate the gut microbiota suggests a potential mechanism through which they contribute to metabolic health [21]. Kaempferol, a bioactive compound abundant in haskap berry leaves, contributes to the potential of these leaves to mitigate inflammatory processes associated with diabetes. This flavonoid presents an additional layer of support against chronic inflammation, a key factor in developing and progressing diabetes-related complications. Studies have highlighted the anti-inflammatory properties of kaempferol, suggesting its ability to modulate pathways involved in inflammation. Chronic inflammation is closely linked to the pathogenesis of diabetes and its complications, making compounds with anti-inflammatory effects valuable in managing the condition [22,23]. In the context of haskap berry leaves, the presence of kaempferol adds to the overall anti-inflammatory profile of the phytochemical composition.

By targeting inflammatory processes, kaempferol may help alleviate the impact of inflammation on insulin sensitivity and glucose homeostasis, contributing to better metabolic health. The antioxidant properties of myricetin contribute to the overall antioxidant capacity of haskap berry leaf extracts. Antioxidants are crucial in neutralising reactive oxygen species (ROS) and mitigating oxidative stress, a critical factor in the pathogenesis of diabetes and its complications [24,25]. By scavenging free radicals, myricetin may help protect cells from oxidative damage, offering a layer of defence against diabetes-related oxidative stress. Furthermore, myricetin’s potential in cardiovascular protection aligns with the need for comprehensive strategies for managing diabetes. Diabetes is often accompanied by an increased risk of cardiovascular issues, making compounds with cardiovascular benefits valuable in therapeutic approaches [26]. The collective activity of these polyphenolic compounds in haskap berry leaves extends beyond individual benefits, providing a comprehensive approach to diabetes management. Their synergistic effects counteract oxidative stress, address chronic inflammation, and contribute to cardiovascular health. Additionally, the potential modulation of the gut microbiota composition suggests an avenue for influencing metabolic processes relevant to diabetes [27,28,29].

In our study, dextran, a group of polysaccharides with prebiotic potential, serves as an ideal carrier for the bioactive compounds in haskap berry leaf extracts. Its non-digestible nature ensures targeted delivery to the colon, promoting the growth of probiotic strains like *Bifidobacterium* and lactobacilli [30,31]. Moreover, dextran’s role as a carrier offers advantages by encapsulating and protecting sensitive bioactive compounds from degradation, ensuring their bioavailability in the gut [32,33].

This comprehensive investigation aims to unveil the multifaceted potential of haskap berry leaf-based dextran formulations in enhancing gut health and overall well-being, particularly in diabetes. Integrating phytochemical analysis, enzymatic inhibition assays, and prebiotic assessments contributes to a holistic understanding of the health-promoting properties of haskap berry leaf extracts, with potential implications for diabetes management.

In the current study, as we focus on haskap berry leaves as the source material for extract preparation, analysing their composition, antioxidant capacity, and inhibitory potential against key enzymes marks a crucial step. The selection of the most promising extract for pre-formulation with different dextrans of varying chain lengths points towards the necessity of developing systems with the potential to modulate gut microbiomes, especially concerning metabolic diseases like diabetes.

## 2. Materials and Methods

### 2.1. Materials

Standards of the determined substances, loganic acid, chlorogenic acid, caffeic acid, quercetin, and rutin were obtained from Sigma-Aldrich (St. Louis, MO, USA).

Prebiotic carriers in the form of dextran with different chain weights, 5000, 40,000, and 70,000 were purchased from Chemat (Poland, Gdańsk)

Reagents used in conducted studies, α-D-glucopyranoside (PNPG), α-glucosidase, acarbose, 2,2-Diphenyl-1-picrylhydrazyl, TPTZ (2,4,6-tripyridyl-S-triazine), iron (III) chloride hexahydrate (FeCl_3_ × 6H_2_O), Folin–Ciocalteu’s phenol reagent, sodium carbonate, 2,4,6-tris(2-pyridyl)-1,3,5-triazine (TPTZ, C_18_H_12_N_6_), iron(III) chloride hexahydrate (FeCl_3_·6H_2_O), sodium chloride, bovine serum, hexadecyltrimethylammonium bromide (CTAB), hyaluronic acid (HA), pancreatic lipase, Tris-HCL buffer, para-Nitrophenylphosphate (pNPP), Triton-X, sodium deoxycholate, and gum Arabic were supplied by Sigma-Aldrich, St. Louis, MO, USA. Methanol, isopropanol, and acetone (Super Purity Solvent, Methanol 215 SPS) were supplied by ROMIL Ltd., Cambridge, UK.

High-quality pure and ultra-high-quality pure water was prepared using a Direct-Q 3 UV Merck Millipore (Burlington, MA, USA) purification system.

### 2.2. Plant Material

The research material came from an industrial plantation located in Moszna, near Nałęczów, Poland. The plantation, with an area of 2 ha and dimensions of 40 m × 500 m, was established in 2017. Before establishing the plantation, the soil was enriched with 30 tons of horse manure. On the plantation, ten rows of haskap berry seedlings of the Jugana variety, purchased from a local producer, were planted. In order to reduce weed infestation, the plantation was covered with agro textiles. Irrigation lines were run along the bush lines, supplied with water from a deep well. Haskap berry plants are nourished yearly in spring with the multi-component Florovit universal fertiliser (1:400 solution). The plants are naturally sprinkled with sawdust every year to acidify the soil.

### 2.3. Extraction Method

The extraction process was carried out using the previously used methodology described by the authors, with minor changes [9,34]. In order to optimise the extraction process, a change of the extractant was proposed:

- Extract 1: 0% EtOH, 100% H_2_O
- Extract 2: 25% EtOH, 75% H_2_O
- Extract 3: 50% EtOH, 50% H_2_O
- Extract 4: 75% EtOH, 25% H_2_O
- Extract 5: 98% EtOH, 0% H_2_O

The extractions were filtered under reduced pressure to obtain the supernatant used for the analysis. The extract, prepared this way, was divided into small portions and frozen at −20 °C to maintain its quantitative and qualitative composition throughout this research.

### 2.4. Analysis of the Content of Bioactive Compounds

To analyse the content of bioactive compounds in the prepared extracts and to develop dextran systems, the authors used a previously described HPLC method and TPC assay [9].

An HPLC method using a UHPLC Nexera system (Shimadzu, Kyoto, Japan) with a Zorbax SB-C18 column (4.6 × 100 mm, 3.5 Micron) was employed to analyse phenolic compounds (loganic acid, chlorogenic acid, caffeic acid, rutin, and quercetin). The mobile phase (A: 0.1% acetic acid, B: acetonitrile) followed a gradient elution (%B): 0 min, 10%; 30 min, 20%; 60 min, 30%; 70 min, 45%; 70.1 min, 10%, with a 10 min column wash. Detection occurred at 210, 240, 254, 270, 320, and 380 nm.

Total phenolic content (TPC) was determined using the Folin–Ciocalteu method. A 50 µL plant extract solution (20 times diluted) was mixed with 50 µL Folin–Ciocalteu reagent and 100 µL distilled water. After pre-incubation (5 min, 37 °C, 100 rpm), 100 µL 20% Na_2_CO3 aq. solution was added, followed by incubation (30 min, 37 °C, 100 rpm). Absorbance was read at 750 nm against the water blank in six replicates. TPC was expressed as mg of gallic acid equivalent (GAE) per g of dry leaf mass using a gallic acid standard curve (y = 10.123 − 0.3142; R^2^ = 0.9973, 0.06–0.2 mg/mL). The TPC content in the tested extract was calculated based on the gallic acid standard curve (Supplementary Materials, Figure S1).

### 2.5. Dextran System Preparation

The preparation of the dextran system involved several sequential steps. Initially, extracts were obtained and evaporated to dryness in a rotary evaporator. Simultaneously, a 100 mL 20% dextran solution was prepared in a separate flask. This step was performed independently three times, each time varying the molecular weight of the dextran chain, as follows: system 1 (dextran 5000), system 2 (dextran 40,000), and system 3 (dextran 70,000). The prepared dextran solution was then added to the flask containing the dried extract and the mixture was stirred until complete dissolution. This crucial step ensured the incorporation of the dextran compound into the extract. The resulting flask was frozen at −20 °C and subjected to lyophilisation. Lyophilization, a freeze-drying process, removed the frozen solvent from the mixture, leaving behind a stable and dry dextran system. After preparation, the systems were homogenised using an agate mortar and the prepared samples were stored in tightly closed Falcon tubes at −20 °C, additionally protected against moisture with parafilm.

### 2.6. Biological Activity

In order to evaluate antioxidant capacity, the ABTS, CUPRAC, DPPH, and FRAP inhibition assays were determined according to the previously reported methods [35,36].

ABTS Assay:

ABTS cation radicals were generated by reacting ABTS with potassium persulfate. Subsequently, 10.0 μL of sample solutions were combined with 200.0 μL of ABTS•+ solution in 96-well plates. After 10 min of room temperature incubation with shaking, absorbance was measured at λ = 734 nm. ABTS scavenging activity was calculated using the following formula:

ABTS scavenging activity (%) = (A0 − A1)/A0 × 100%

Trolox served as the standard (Figure S2).

CUPRAC Assay:

Neocuproine and copper ion (II) complex was interacted with the sample solution to evaluate its antioxidant potential. A CUPRAC reagent, composed of acetate buffer, neocuproine, and CuC_l2_·H_2_O, was prepared. Subsequently, 50.0 µL of system solutions and 150.0 µL of the CUPRAC reagent were applied to a 96-well plate and incubated for 30 min at room temperature. Absorbance was measured at 450 nm and the results were expressed as Trolox equivalents (Figure S3).

DPPH Assay:

Aqueous solutions were mixed with DPPH solution and the reaction mixture was incubated in the dark. Absorbance was measured at 517 nm. DPPH scavenging activity was calculated using the following formula:

DPPH scavenging activity (%) = (A0 − A1)/A0 × 100%

The results were expressed as Trolox equivalents (Figure S4).

FRAP Assay:

The FRAP assay involved mixing tested extracts with FRAP reagent and incubating the mixture at 37 °C. Absorbance was read at 593 nm and the results were expressed as Trolox equivalents (Figure S5).

Further enzymatic inhibition activity against α-glucosidase, hyaluronidase, and lipase was carried out following previously described methods for extracts and dextran systems [9,37].

A modified spectrophotometric method, as described by Telagari et al. [38], was used to assess the inhibition of α-glucosidase by haskap berry leaves water extracts. Briefly, 50.0 μL of sample solution (concentration ranges specified for each extract) or acarbose (positive control, 1.0–5.0 mg mL^−1^) was pre-incubated with 50.0 μL of 0.1 M phosphate buffer (pH 6.8) and 30.0 µL α-glucosidase solution (1.0 U mL^−1^) at 37 °C for 15 min. Subsequently, 20.0 μL of 5 mM p-nitrophenyl-α-D-glucopyranoside (pNPG) solution was added and incubated at 37 °C for 20 min. The reaction was halted by adding 100.0 µL of 0.2 M sodium carbonate. Absorbance was measured at 405 nm.

For lipase inhibition, a modified method by Lewis and Liu was employed. Plant extracts Orlistat (positive control; 50.0 µg/mL) or water (negative control) were added to 96-well plates [39]. Pancreatic lipase solution (10.0 mg/mL), enzyme solution, and substrate solution (pNPP in isopropanol) were sequentially added and the absorbance was measured at 405 nm.

Hyaluronidase inhibition was determined using a modified method by Grabowska et al. [40]. Incubation buffer, enzyme, plant extracts, and HA solution were mixed and incubated. After incubation, cetrimonium bromide was added and turbidity was measured at 600 nm. Quercetin served as the positive control.

### 2.7. Evaluation of Prebiotic Potential

Cultures of regenerated probiotic and potentially pathogenic strains underwent centrifugation at 4500× *g* for 10 min and cells were subsequently washed with sterile physiological saline (Table 1). The cell suspensions were then adjusted to 0.5 on the McFarland scale (1.5 × 10^8^ CFU/mL), using the McFarland Densitometer (Biosan). These adjusted cell suspensions were employed for inoculating 2 mL of standard MRS broth medium (used as the control) and MRS medium supplemented with a single-component system consisting of dextran, inulin, and trehalose, as well as a system incorporating these dextrans. The abovementioned components were incorporated into the MRS medium at a mass ratio of 1%. The initial bacterial concentration in all media was standardised to 1.5 × 10^5^ CFU/mL. Incubation was carried out without pH regulation at 37 °C for 24 h. Subsequently, the viable cell count was determined using Koch’s plate method. For this, collected samples were subjected to decimal dilution in sterile physiological saline, plated on Petri dishes with MRS agar (OXOID), and then incubated at 37 °C for 48–72 h. The colonies, used for assessing viable cell numbers, were enumerated using an automatic colony counter (Easy Count 2) and expressed in CFU/mL. The strain that exhibited the most favourable response to the tested/obtained systems was cultured on a 200.0 mL scale for 96 h under the same conditions. Throughout the incubation period, samples were taken at 12, 24, 48, and 72 h to evaluate the viable cell count. The results were reported as colony-forming units (CFU/mL). All tests were conducted in triplicate to ensure reliability.

### 2.8. Statistical Analysis

Statistica 13.3 software (StatSoft Poland, Krakow, Poland) was used for the statistical analysis. Experimental data were analysed using the skewness and kurtosis tests to determine the normality of each distribution and Levene’s test assessed the equality of variances. Statistical significance was determined using a one-way analysis of variance (ANOVA) followed by the Bonferroni post hoc test (to compare the experimental results between each extract and obtained systems in antioxidant and enzyme assays; in prebiotic potential studies, statistical analysis was performed for each time point and each strain, where the results obtained for each system or probiotic were compared to control (0)). Differences were considered significant at *p* < 0.05.

## 3. Results and Discussion

The investigation into the potential health benefits of haskap berry leaves, particularly in diabetes management, has yielded promising findings across multiple facets. The rich phytochemical composition of haskap berry leaves, characterised by a diverse array of polyphenolic compounds, has been a focal point of research due to its multifaceted impact on various aspects of diabetes. One notable aspect is the antioxidant properties inherent in these polyphenols. The potent antioxidant capacity of haskap berry leaves, attributed to compounds such as quercetin, rutin, catechins, kaempferol, and myricetin, suggests a potential role in mitigating oxidative stress—a critical factor in the development and progression of diabetes-related complications. By reducing oxidative stress, haskap berry leaves may offer protective effects against complications associated with diabetes, contributing to an overall improvement in health outcomes.

Moreover, the investigated polyphenols in haskap berry leaves, including quercetin and rutin, have demonstrated antidiabetic properties. These compounds exhibit the potential to enhance insulin sensitivity, regulate glucose homeostasis, and reduce blood glucose levels. Such effects are particularly relevant in type 2 diabetes, where insulin resistance and impaired glucose metabolism are central features. The ability of haskap berry leaves to modulate these factors underscores their potential as a natural intervention for managing type 2 diabetes. [9,11,41]. These studies comprehensively explore the extracts and dextran systems obtained from the phytochemical analysis, enzymatic inhibition assays, and subsequent assessments on the potential modulation of the gut microbiome. The intricate interplay between the rich polyphenolic composition of haskap berry leaves and the prebiotic carrier, dextran, forms the foundation of our endeavour to unlock their synergistic potential for enhancing gut health and addressing the metabolic imbalances associated with diabetes.

The extraction process, conducted following the established methodology with minor modifications, aimed to optimise the extraction of bioactive compounds from haskap berry leaves [9]. The proposed variations in the extractant composition, ranging from Extract 1 with 0% EtOH and 100% H_2_O to Extract 5 with 98% EtOH and 0% H_2_O, were designed to assess the impact of solvent polarity on the extraction of bioactive constituents. This systematic approach allowed us to explore the spectrum of compounds extracted under different solvent conditions and to understand the influence of polarity on the extraction yield. It is noteworthy to mention that the infusions of haskap berry leaves were previously investigated in an earlier publication. In this study, our focus shifted towards understanding how altering the extractant composition would translate into changes in the extract’s composition and activity. By examining the impact of different solvents on the extraction process, we aimed to elucidate the role of solvent polarity in shaping the phytochemical profile of the extracts. This complementary investigation provides valuable insights into the extraction dynamics and enhances our understanding of how varying extraction conditions influences the bioactive constituents and overall activity of haskap berry leaf extracts.

The results of the phytochemical analysis reveal the diverse composition of haskap berry leaf extracts, with a focus on total polyphenolic content (TPC) and specific bioactive compounds, including loganic acid, chlorogenic acid, caffeic acid, rutin, and quercetin. These findings hold implications for the potential health benefits of haskap berry leaves, particularly in diabetes management (Table 2).

The TPC values obtained for the five extracts ranged from 51.057 to 60.014 mg/g DL. The observed variations could be attributed to the extraction process. Notably, Extract 3 consistently exhibited the highest TPC, suggesting the presence of optimal conditions for polyphenol extraction. One noteworthy compound in all extracts is loganic acid, a secoiridoid glycoside known for its antioxidant properties. Concentrations ranged from 2.754 to 2.982 mg/g DL, showing relatively stable levels across different extraction conditions. The antioxidant potential of loganic acid aligns with its potential health benefits and further research could explore its specific role in mitigating oxidative stress, particularly in the context of diseases such as diabetes [42]. Chlorogenic acid, a phenolic compound associated with various health benefits, exhibited concentrations ranging from 0.956 to 1.127 mg/g DL. Extract 3, with the highest concentration, emerges as a potential source of this compound. Given the reported health benefits of chlorogenic acid, one of the mechanisms by which chlorogenic acid may exert its beneficial effects in diabetes is via its ability to inhibit certain enzymes involved in glucose metabolism. Specifically, chlorogenic acid has been shown to inhibit α-glucosidase and α -amylase, enzymes responsible for breaking down carbohydrates into glucose in the digestive system [43,44]. By slowing down the digestion and absorption of carbohydrates, chlorogenic acid may help prevent rapid spikes in blood sugar levels after meals.

Additionally, chlorogenic acid has improved insulin sensitivity [45,46]. The reported findings suggest that chlorogenic acid may exert positive effects at the cellular level, enhancing insulin action and facilitating glucose uptake by various tissues. This improvement in insulin sensitivity holds significant implications, particularly in the context of Type 2 diabetes, where insulin resistance is a central pathology feature [47]. The cellular mechanisms underlying the observed enhancement in insulin sensitivity are multifaceted. Chlorogenic acid may modulate signalling pathways associated with insulin action, potentially influencing critical components involved in glucose metabolism. By facilitating glucose uptake by tissues, chlorogenic acid contributes to improved insulin responsiveness, thereby addressing a crucial aspect of the dysregulated glucose homeostasis characteristic of Type 2 diabetes [48]. The documented impact of chlorogenic acid on insulin sensitivity aligns with its broader antidiabetic properties. Beyond its antioxidant and anti-inflammatory attributes, the ability to positively influence insulin responsiveness positions chlorogenic acid as a bioactive compound with comprehensive metabolic implications. These findings underscore the potential therapeutic relevance of chlorogenic acid in managing Type 2 diabetes, pointing towards its multifaceted role in ameliorating the complex pathophysiology of the condition [46,49].

Moreover, chlorogenic acid has antioxidant properties, which can help to neutralise harmful free radicals in the body [50]. Oxidative stress is a recognised contributor to the development and progression of complications related to diabetes. By its antioxidant properties, chlorogenic acid emerges as a potential mitigator of oxidative stress and a protective agent against diabetes-related complications. The multifaceted antioxidant mechanisms of chlorogenic acid involve scavenging free radicals, inhibiting the formation of reactive oxygen species (ROS), and modulating antioxidant enzyme activities [51]. In the context of diabetes, where heightened oxidative stress is implicated in vascular complications, nephropathy, retinopathy, and neuropathy, the antioxidative actions of chlorogenic acid gain particular significance. Studies have suggested that chlorogenic acid’s ability to counteract oxidative stress contributes to preserving cellular integrity and function, ultimately attenuating the risk of diabetic complications. The protective effects of chlorogenic acid extend to its influence on inflammatory pathways, further supporting its potential to avert complications associated with diabetes [52,53]. By modulating oxidative stress and inflammation, chlorogenic acid presents a holistic approach to mitigating the intricate network of events leading to diabetic complications [54,55]. It is important to note that while research indicates the potential benefits of chlorogenic acid in diabetes, more studies are needed to understand its impact and determine optimal dosages fully.

Recent studies have illuminated the intricate interplay between dietary polyphenols, chlorogenic acid, and the gut microbiota. Chlorogenic acid in the gut may act as a substrate for specific beneficial bacteria, fostering their growth and potentially enriching these microbial populations [56]. The resulting modulation of the gut microbiota composition is particularly interesting due to its association with metabolic functions and overall health. The capacity of chlorogenic acid to selectively stimulate the proliferation of beneficial bacteria aligns with the broader concept of prebiotics, substances that promote the growth and activity of beneficial microorganisms. This prebiotic-like potential of chlorogenic acid suggests a dual mechanism, through which it may confer health benefits—directly, through its antioxidant and anti-inflammatory properties and indirectly, by shaping the gut microbiota to support metabolic well-being [57,58]. However, it is essential to acknowledge the complexity of the gut microbiome and the need for further investigations to delineate the specific strains influenced by chlorogenic acid and the underlying mechanisms. Elucidating these details is crucial for advancing our understanding of the intricate interplay between dietary components, the gut microbiota, and metabolic health.

Caffeic acid, known for its antioxidant and anti-inflammatory effects, showed concentrations from 0.077 to 0.097 mg/g DL [59,60,61]. Its known antioxidant and anti-inflammatory effects are relevant in the context of diabetes, where these properties could play a role in mitigating inflammation and oxidative stress. Rutin and quercetin, flavonoids with diverse health-promoting effects, demonstrated concentrations ranging from 0.871 to 1.157 mg/g DL and 1.249 to 1.509 mg/g DL, respectively. These compounds have been studied for their potential effects on various health conditions, including diabetes [62,63]. Both rutin and quercetin are known for their potent antioxidant properties [64,65]. Antioxidants help to neutralise free radicals in the body, which are molecules that can cause oxidative stress and damage cells. Oxidative stress is implicated in the development and progression of diabetes and its complications [66]. Chronic inflammation is closely linked to developing insulin resistance and diabetes [67]. Rutin and quercetin have demonstrated anti-inflammatory effects in various studies [68,69].

The variations among extracts also open avenues for further research to optimise extraction methods to enhance the yield of specific bioactive compounds. Extract 3, with its consistently higher concentrations across multiple components, emerges as a promising candidate for further exploration in developing functional foods or nutraceuticals targeting health, especially in individuals with diabetes. The potential bioactivity of the identified compounds, such as loganic acid: 2.974 mg/g, chlorogenic acid: 1.125 mg/g, caffeic acid: 0.083 mg/g, rutin: 1.137 mg/g, and quercetin: 1.501 mg/g, warrants in-depth investigations to elucidate their specific roles in mitigating diabetes-related complications and promoting overall health.

**Figure 1:**
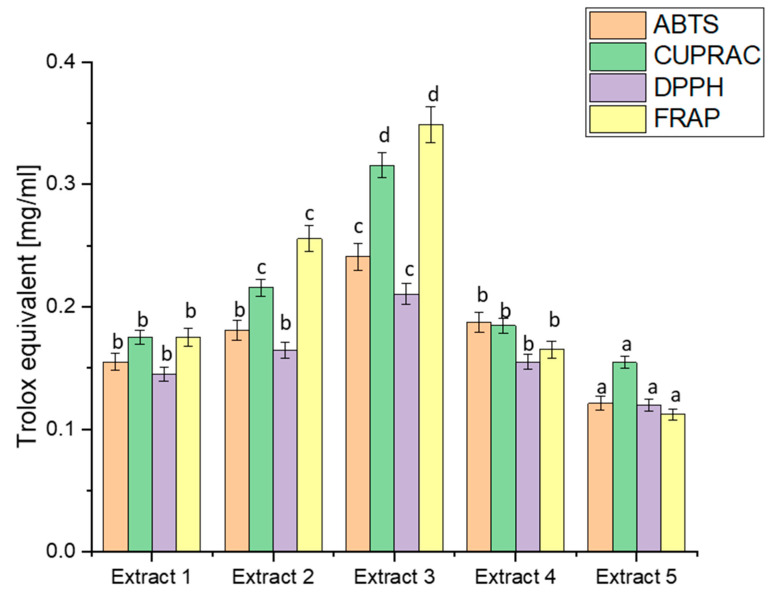
Antioxidant potential of extracts obtained from haskap berry leaves expressed as Trolox equivalent [mg/mL]. Data expressed as mean ± SD; columns with different superscript letters (a–d) differ significantly (*p* < 0.05).

The analysis of biological activity began with determining the antioxidant potential, which is an indispensable basis for the activity of plant raw materials using four widely recognised in vitro models: ABTS, CUPRAC, DPPH, and FRAP [70]. The diverse methodologies employed by these assays offer a comprehensive evaluation of the antioxidant potential of haskap berry extracts, shedding light on their efficacy in different aspects of radical scavenging and metal-reducing activities (Figure 1).

A comparison of the antioxidant capacity of the obtained extracts indicated the significantly best free radical scavenging ability of Extract 3. The ABTS values ranged from 0.1212 to 0.2412, indicating diverse antioxidant capacities across extracts. Extract 3 exhibited the highest ABTS activity, correlating with its elevated TPC. This aligns with the general understanding that polyphenols, such as those in haskap berry leaves, contribute significantly to ABTS scavenging activity [71]. CUPRAC values varied from 0.1545 to 0.3156, with Extract 3 displaying the highest activity again. The notable correlation between high CUPRAC values and increased concentrations of bioactive compounds, especially flavonoids like rutin and quercetin, reinforces the potential of haskap berry leaves in copper reduction-based assays [72]. DPPH values ranged from 0.1200 to 0.2105, with Extract 3 showing the highest radical scavenging activity. This aligns with the previously discussed polyphenolic composition, emphasising the contributions of compounds like chlorogenic acid, rutin, and quercetin to DPPH radical scavenging [73]. FRAP values spanned from 0.1121 to 0.3488, with Extract 3 again demonstrating the highest ferric-reducing activity. The parallelism between FRAP results and the polyphenolic composition underscores the potential role of compounds like loganic acid and chlorogenic acid in electron transfer-based antioxidant assays [74]. The observed trends in antioxidant capacity align with the phytochemical composition discussed earlier. Extract 3 consistently had the highest TPC, loganic acid, chlorogenic acid, rutin, and quercetin concentrations. This suggests a direct correlation between the polyphenolic content and the antioxidant potential of haskap berry leaf extracts. The consistent trends in antioxidant capacity across different assays, particularly the prominence of Extract 3, hold substantial health implications, especially in managing diabetes.

**Figure 2:**
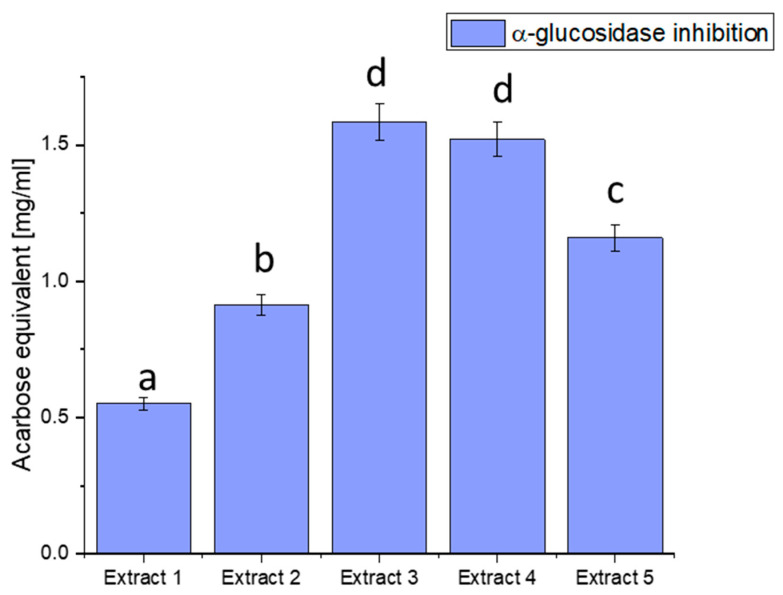
Assessment of the ability to inhibit α-glucosidase by haskap berry leaf extracts expressed as acarbose equivalent [mg/mL]. Data expressed as mean ± SD; columns with different superscript letters (a–d) differ significantly (*p* < 0.05).

We focused on three glucosidase, hyaluronidase, and lipase inhibitions of enzymes essential to health, particularly in metabolic disorders like diabetes. α-glucosidase is an enzyme that breaks down complex carbohydrates into simpler sugars, such as glucose. Excessive activity of this enzyme can lead to a rapid increase in blood glucose levels, a significant factor in the pathogenesis of diabetes. Our study aimed to assess the ability of haskap berry leaf extracts to inhibit the activity of α-glucosidase, potentially influencing the control of blood glucose levels (Figure 2).

The observed discrepancies in α-glucosidase inhibition can be attributed to the diverse phytochemical composition of the extracts. Extract 3 demonstrated the most potent inhibitory effect, distinguished by elevated concentrations of bioactive compounds such as quercetin, rutin, and chlorogenic acid. These compounds are recognised for modulating enzyme activity, particularly in glucose metabolism.

Plant-derived compounds, rich in bioactive constituents, have demonstrated significant potential as inhibitors of α-glucosidase, offering a natural approach to managing glucose metabolism. The results obtained from the haskap berry leaf extracts in this study, with Extract 3 exhibiting the highest α-glucosidase inhibitory activity, contribute to the broader understanding of the therapeutic potential of plant-based inhibitors. The observed α-glucosidase inhibition aligns with previous research highlighting the efficacy of plant-derived compounds in modulating enzyme activity [75]. The diverse phytochemical composition of plants, including flavonoids, polyphenols, and other secondary metabolites, has been associated with inhibitory effects on α-glucosidase. Extract 3, rich in quercetin, rutin, and chlorogenic acid, compounds known for their antidiabetic properties, showcases the potential synergy of these bioactive constituents in inhibiting α-glucosidase. The use of plant-derived α-glucosidase inhibitors in therapeutics presents several advantages. Firstly, these inhibitors may offer a more holistic and multi-targeted approach than conventional medications. The diverse bioactive compounds in plant extracts may act on various pathways involved in glucose metabolism, potentially mitigating side effects associated with single-target drugs. Secondly, the natural origin of these inhibitors aligns with the growing interest in plant-based medicines, emphasising safety and sustainability [76,77].

Furthermore, the inhibitory effects observed in haskap berry extracts, particularly Extract 3, highlight the potential for developing functional foods or nutraceuticals to manage conditions like diabetes. Incorporating such natural inhibitors into the diet may provide a practical and palatable means of supporting metabolic health. However, it is essential to consider factors such as bioavailability, dosage, and long-term effects in developing these interventions.

**Figure 3:**
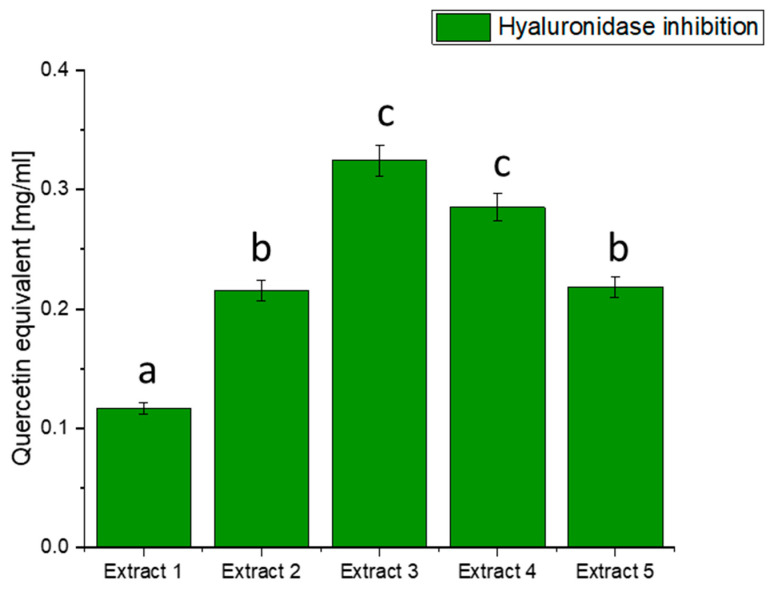
Assessment of the ability to inhibit hyaluronidase by haskap berry leaf extracts expressed as quercetin equivalent [mg/mL]. Data expressed as mean ± SD; columns with different superscript letters (a–c) differ significantly (*p* < 0.05).

Hyaluronidase is an enzyme involved in the degradation of hyaluronic acid, a crucial component of the extracellular matrix. Imbalances between the synthesis and degradation of hyaluronic acid can contribute to inflammation and related conditions, including diabetes [78]. Our investigation sought to understand whether extracts from haskap berry leaves could exhibit inhibitory activity against hyaluronidase, potentially offering benefits in controlling inflammatory processes associated with diabetes (Figure 3).

The results of hyaluronidase inhibition indicate varying degrees of activity across the different extracts, with Extract 3 exhibiting the highest potency in terms of quercetin equivalent. The observed inhibition of hyaluronidase by haskap berry leaf extracts, mainly Extract 3, which is rich in quercetin, suggests a potential anti-inflammatory effect. Quercetin, a flavonoid with known anti-inflammatory properties, may contribute to mitigating the inflammatory processes associated with diabetes [79]. The anti-inflammatory potential of quercetin, as indicated by the hyaluronidase inhibition, aligns with the broader understanding of its effects on inflammatory pathways [80]. Including haskap berry leaf extracts in the diet could be explored as a complementary strategy to mitigate the inflammatory component of diabetes beyond glycaemic control alone. It is important to note that while the in vitro inhibition of hyaluronidase is promising, further studies, including in vivo investigations and clinical trials, are needed to validate these findings and establish the relevance of haskap berry leaf extracts, especially Extract 3, in managing diabetes-associated inflammation.

**Figure 4:**
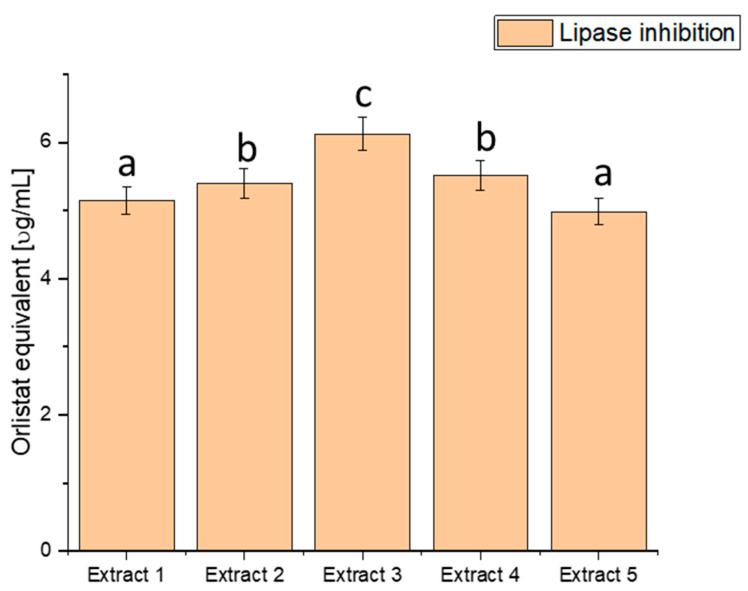
Assessment of the ability to inhibit lipase by haskap berry leaf extracts expressed as orlistat equivalent [µg/mL]. Data expressed as mean ± SD; columns with different superscript letters (a–c) differ significantly (*p* < 0.05).

Lipase is an enzyme responsible for digesting fats in the body. Excessive lipase activity can lead to the uncontrolled breakdown of fats, which, in turn, may be linked to obesity and insulin resistance, both risk factors for diabetes. We explored the ability of haskap berry leaf extracts to inhibit lipase activity, potentially influencing the control of lipid metabolism-related processes (Figure 4).

The assessment of lipase inhibition, expressed in orlistat equivalent (µg/mL), reveals distinct inhibitory potential across the various extracts. Extract 1 exhibited an inhibitory effect of 5.151 ± 0.20456, Extract 2 showed 5.401 ± 0.21448, Extract 3 demonstrated the highest potency with 6.127 ± 0.24332, Extract 4 had an inhibitory effect of 5.515 ± 0.21901, and Extract 5 exhibited 4.987 ± 0.19804. These values showcase a range of inhibitory effects on lipase activity, with Extract 3 demonstrating the highest potency, as indicated by the highest orlistat equivalent value. The heightened inhibitory effect on lipase activity, particularly in Extract 3, suggests a more substantial potential to modulate fat absorption. Controlling postprandial hyperlipidaemia is vital in managing diabetes and reducing the risk of associated cardiovascular issues [81,82]. Extract 3, with its superior lipase inhibition, emerges as a promising natural source for developing interventions to regulate lipid metabolism in individuals with diabetes. By curbing the breakdown of dietary fats, haskap berry extracts, significantly Extract 3, could improve lipid control and overall metabolic health.

However, it is crucial to note that these findings are derived from in vitro assays and their translation to clinical applications requires further scrutiny. Rigorous clinical trials and in vivo studies are imperative to validate the effectiveness and safety of haskap berry extracts, mainly Extract 3, in managing lipid metabolism among individuals with diabetes.

To harness the potential of haskap berry leaves for developing a potentially beneficial system for gut health, a crucial phase of our study involved preparative work to formulate a synergistic blend with dextran. The choice of an optimal haskap berry leaf extract for this dextran formulation was guided by our earlier findings, with Extract 3 (loganic acid: 2.974 mg/g, chlorogenic acid: 1.125 mg/g, caffeic acid: 0.083 mg/g, rutin: 1.137 mg/g, and quercetin: 1.501 mg/g) emerging as the most active and promising candidate. The decision to focus on Extract 3 for further investigations and dextran formulations was grounded in its exceptional performance across multiple parameters in our preliminary studies. This extract exhibited the highest TPC, robust antioxidant capacity, and significant inhibitory potential against key enzymes associated with diabetes, namely α-glucosidase (1.4915 mg/mL, expressed as acarbose equivalent), hyaluronidase (0.2982 mg/mL, expressed as quercetin equivalent), and lipase (5.8715 µg/mL, expressed as orlistat equivalent).

Dextran systems were obtained by incorporating Extract 3 from haskap berry leaves and dextran as a prebiotic carrier. In the initial stage, raw haskap berry leaves underwent a solvent extraction process to obtain the base extracts. These extracts formed the foundation for further formulating systems to modulate the gut microbiome. The extracts were combined with dextran of varying molecular weights, namely 5000, 40,000, and 70,000 g/mol, and were further lyophilised to produce the final systems.

Table 3 compares the content of bioactive compounds in the prepared dextran systems (System 1, System 2, and System 3) to the pure haskap berry leaves extract. The measured values for TPC and individual bioactive compounds are below.

The meticulous analysis of the active compound content in the prepared dextran systems yields promising insights into the robustness of the formulations. This uniformity indicates that integrating dextran during the preparative work did not compromise the overall polyphenolic richness, a critical aspect considering the well-established health benefits associated with polyphenols. The stability of individual bioactive compounds, including loganic acid, chlorogenic acid, caffeic acid, rutin, and quercetin, further underscores the resilience of the dextran formulations. Minimal variations in the concentrations of these bioactive compounds suggest that the preparative process did not adversely impact their structural integrity.

Evaluating the prepared dextran systems’ antioxidant potential and biological activity (System 1, System 2, and System 3) is crucial for understanding their potential health benefits. Additionally, these activities are compared with the reference Extract 3, to assess the impact of the dextran formulations on these essential parameters (Table 4).

The ABTS scavenging activity ranged from 0.2312 to 0.2514 mg/mL, with System 2 exhibiting the highest value. Despite slight variations, all dextran systems and Extract 3 displayed comparable antioxidant potential. System 2 demonstrated the highest cupric ion-reducing capacity at 0.3215 mg/mL. Again, the dextran systems closely mirrored the antioxidant potential of Extract 3. DPPH radical scavenging activity ranged from 0.2005 to 0.2245 mg/mL. System 2 displayed the highest activity, reinforcing that dextran formulations maintain robust antioxidant potential. The ferric-reducing ability varied from 0.3288 to 0.3647 mg/mL, with System 2 exhibiting the highest value. Extract 3, once more, demonstrated a similar range of antioxidant potential.

All dextran systems exhibited inhibition of α -glucosidase, with System 2 showing the highest activity at 1.5541 mg/mL. While Extract 3 had slightly higher values, the dextran systems demonstrated noteworthy α-glucosidase inhibitory potential. System 2 displayed the highest inhibition of hyaluronidase at 0.3144 mg/mL. Compared to Extract 3, the dextran systems maintained substantial inhibitory effects on hyaluronidase activity. The dextran systems demonstrated effective lipase inhibition, with System 2 showing the highest activity at 6.1112 µg/mL. Extract three and the dextran systems showcased comparable potential in inhibiting lipase activity.

The microbiological analysis, presented in Table 5, provides a comprehensive insight into the dynamic interactions between various bacterial strains and the dextran systems under investigation. This exploration delves into the microbial responses over 72 h, shedding light on the proliferation patterns and potential prebiotic effects exerted by the tested substrates. The intricate interplay between prebiotic carriers, such as dextran, and different bacterial species is unveiled, offering valuable information for understanding the nuanced dynamics of these interactions.

The results of the microbiological analysis shed light on the intricate interactions between the formulated dextran systems and specific bacterial strains, particularly those belonging to the *Bifidobacterium* and lactobacilli genera. Notably, *Bifidobacterium longum, Bifidobacterium animalis, Lgb. salivarius, Lab. plantarum* 299v, *Lab. helveticus*, and *Lab. rhamnosus* GG exhibited varying degrees of proliferation in response to the dextran systems. These bacterial strains are of particular significance in diabetes, given their potential roles in metabolic health. *Bifidobacterium* and *Lab.* species are recognised for their ability to ferment dietary fibres and produce short-chain fatty acids (SCFAs), which have been implicated in various metabolic processes. SCFAs, especially butyrate, have anti-inflammatory effects, improve insulin sensitivity, and contribute to glucose homeostasis. The increased abundance of *Bifidobacterium longum* and *Bifidobacterium animalis* in response to the dextran systems is noteworthy. *Bifidobacteria* are known for their bifidogenic effects, promoting the growth of beneficial bacteria while inhibiting pathogenic species. This bifidogenic activity is particularly relevant in diabetes, where maintaining a balanced gut microbiota is crucial for mitigating inflammation and supporting metabolic function.

Similarly, the proliferation of lactobacilli species, including *Lgb. salivarius, Lab. plantarum* 299v, *Lab. helveticus*, and *Lab. rhamnosus* GG, is of interest. *Lactobacilli* are associated with producing SCFAs, modulation of gut barrier function, and regulating immune responses. These functions are integral to managing chronic low-grade inflammation, a key factor in diabetes progression and complications.

Incorporating dextran as a prebiotic carrier in the formulations demonstrated notable effects on the proliferation of specific bacterial strains over a 72 h period. The varying responses observed across *Bifidobacterium longum*, *Bifidobacterium animalis*, *Faecalibacterium prausnitzii, Lgb. salivarius, Lab. plantarum 299v, Lab. helveticus*, and *Lab. rhamnosus* GG indicate the potential of dextran in modulating the gut microbiota. In System 2, *Bifidobacterium longum* substantially increased from 2.61 × 10^8^ to 1.49 × 10^9^ after 48 h, suggesting that dextran in this formulation significantly enhanced the proliferation of this beneficial bacterial strain. Similarly, *Bifidobacterium animalis* and *Faecalibacterium prausnitzii* displayed notable growth, reaching 5.65 × 10^8^ and 1.55 × 10^9^, respectively, in System 2 after 48 h, indicating the prebiotic potential of dextran for these strains. *Lgb. salivarius* and *Lab. plantarum* 299v exhibited robust proliferation, particularly in System 2, with values increasing from 1.54 × 10^10^ to 3.31 × 10^10^ and from 1.98 × 10^10^ to 2.99 × 10^10^, respectively, over the 72 h. This underscores the efficacy of dextran as a prebiotic carrier for these specific strains. In contrast, *Lab. helveticus* displayed differential responses across systems, indicating that dextran formulation may influence the growth dynamics of specific bacterial strains. *Lab. rhamnosus* GG exhibited preferential growth in System 2, reaching 2.22 × 10^10^ after 48 h, highlighting dextran composition’s specificity in promoting this probiotic strain’s proliferation.

The microbial analysis over 72 h evaluated the impact of different potentially pathogenic bacterial strains on the control and dextran systems, highlighting essential insights into the prebiotic potential of these formulations. Notably, no statistically significant differences (*p* < 0.05) were observed among the tested conditions, underscoring the importance of a comprehensive examination (Table S1). For assessment, *Bacillus subtilis*, *Enterococcus faecalis*, *Listeria monocytogenes*, *Staphylococcus aureus*, *Escherichia coli*, *Pseudomonas aeruginosa*, and *Salmonella enterica* were explored. The results indicated that dextran in the systems did not exhibit bactericidal activity against these strains, as evidenced by the lack of significant differences compared to the control. Notably, the data revealed that dextran, as a prebiotic carrier, did not stimulate or inhibit the growth of potentially pathogenic bacteria. This finding suggests a neutral interaction, emphasising the safety of dextran in prebiotic formulations. Intriguingly, the absence of bactericidal effects and growth stimulation aligns with the notion of selective prebiotic activity. The formulations did not compromise the viability of beneficial microorganisms, indicating a potential for selective microbial modulation. These results contribute to understanding the complex interactions between prebiotic carriers and bacterial strains. While no direct antimicrobial effects were observed, the absence of growth stimulation supports the hypothesis of selective prebiotic activity, opening avenues for further research to elucidate the specific mechanisms governing these interactions.

The observed responses in bacterial proliferation underscore the critical importance of selecting appropriate prebiotic carriers for the precise modulation of the gut microbiota. The sustained impact of dextran over the 72 h timeframe suggests its viability as a sustained prebiotic substrate and points to its potential for long-term microbiome modulation. This enduring effect aligns with the hypothesis of selective prebiotic activity, as evidenced by the lack of significant bactericidal activity against potentially pathogenic bacterial strains and the absence of growth stimulation.

The need for a deeper exploration of the intricate mechanisms underpinning dextran-mediated prebiotic effects becomes even more apparent in light of these results. Fine-tuning formulations through such investigations can amplify their effectiveness in fostering a harmonious and health-promoting gut microbiome. While the current findings do not indicate direct antimicrobial effects or growth stimulation of potentially pathogenic bacteria, they emphasise the potential of dextran as a neutral or selective prebiotic carrier. In conclusion, these results collectively advocate for the strategic integration of dextran as a prebiotic carrier, highlighting its promise in specifically tailoring the gut microbiota. This underscores its potential applications in developing functional foods or nutraceuticals to enhance gastrointestinal health. The need for further investigations remains apparent, aiming to unravel the nuanced interactions between dextran and diverse bacterial strains, especially in the context of the observed lack of bactericidal activity and growth stimulation against potentially pathogenic bacteria.

## 4. Conclusions

This extensive investigation encompassed a multifaceted exploration into the phytochemical, antioxidant, and prebiotic aspects of haskap berry leaf extracts and their formulated dextran systems. The initial focus on phytochemical composition unravelled a rich tapestry of polyphenols, notably loganic acid, chlorogenic acid, rutin, and quercetin. Extract 3 (loganic acid: 2.974 mg/g, chlorogenic acid: 1.125 mg/g, caffeic acid: 0.083 mg/g, rutin: 1.137 mg/g, and quercetin: 1.501 mg/g) emerged as a powerhouse of these bioactive compounds, aligning with its consistently elevated antioxidant activities across diverse assays, including ABTS: 0.2447 mg/mL, CUPRAC: 0.3121 mg/mL, DPPH: 0.21001 mg/mL, and FRAP: 0.3411 mg/mL, expressed as Trolox equivalent. The subsequent enzymatic inhibition assays unveiled the considerable inhibitory potential of the formulated dextran systems against critical enzymes related to metabolic health. The inhibitory concentrations were determined for α-glucosidase, hyaluronidase, and lipase, expressing the results regarding well-known reference compounds. The inhibition of α-glucosidase, a crucial enzyme involved in carbohydrate digestion, was notable, with an inhibitory concentration of 1.4915 mg/mL expressed as acarbose equivalent. This suggests that the dextran systems have the potential to modulate postprandial glucose levels, making them relevant in the context of conditions like diabetes.

Furthermore, the inhibitory effect on hyaluronidase, with a concentration of 0.2982 mg/mL expressed as quercetin equivalent, highlights the dextran system’s ability to influence the breakdown of hyaluronic acid, an essential component of connective tissues. This inhibition may have implications for managing inflammation and tissue health. The lipase inhibitory activity of the dextran systems was also significant, with a concentration of 5.8715 µg/mL expressed as orlistat equivalent. Lipase plays a crucial role in lipid digestion and the observed inhibition suggests the potential of these dextran formulations in regulating lipid absorption, which could be beneficial in conditions like obesity.

The investigation into the microbiological activity of the formulated dextran systems provided valuable insights into the impact of dextran chain length on specific bacterial strains. The prolonged 72 h analysis revealed distinctive responses among bacterial species to varying dextran molecular weights. *Bifidobacterium longum* exhibited enhanced growth in the presence of dextran 70,000 g/mol, indicating the positive influence of longer dextran chains on the proliferation of this beneficial strain. *Bifidobacterium animalis* also displayed increased growth with dextran 70,000 g/mol, further highlighting the potential of extended dextran chains in promoting the abundance of this probiotic species. In contrast, *Faecalibacterium prausnitzii* preferred dextran 5000 g/mol, showcasing the specificity of bacterial responses to different dextran chain lengths. *Lgb. salivarius* exhibited robust growth across all dextran formulations, suggesting its adaptability to various prebiotic substrates. *Lab. plantarum* 299v displayed notable proliferation with dextran 40,000 g/mol, indicating that a moderate chain length may be optimal for supporting the growth of this probiotic strain. *Lab. helveticus* showed enhanced growth with dextran 40,000 g/mol, emphasising the importance of tailoring dextran formulations to specific bacterial preferences. *Lab. rhamnosus* GG exhibited robust growth across all dextran formulations, suggesting its versatility in utilising different dextran chain lengths as prebiotic substrates. Notably, the lack of significant impact on potentially pathogenic bacterial strains, such as *Bacillus subtilis, Enterococcus faecalis*, *Listeria monocytogenes*, *Staphylococcus aureus*, *Escherichia coli*, *Pseudomonas aeruginosa*, and *Salmonella enterica*, suggests a neutral or selective prebiotic activity. This nuance is crucial in microbiome modulation, presenting an intriguing avenue for further exploration.

Overall, the findings highlight the differential responses of various bacterial strains to distinct dextran molecular weights. Tailoring dextran formulations to the preferences of specific beneficial bacteria, as evidenced in this study, holds promise for optimising gut microbiota modulation strategies to promote overall gastrointestinal health. The successful integration of dextran in dextran systems opens avenues for developing functional foods and nutraceuticals to enhance gastrointestinal health.

## Figures and Tables

**Table 1:** Bacterial strains used in the research, along with an indication of their origin.

Strain Name	Category	Origin
Gram-positive bacteria		
*Bifidobacterium longum*	Probiotic bacteria	ATCC 15707
*Bifidobacterium animalis*	Probiotic bacteria	ATCC BAA-2848
*Faecalibacterium prausnitzii*	Probiotic bacteria	ATCC 29739
*Lactobacillus salivarius*	Probiotic bacteria	ATCC 11741
*Lactiplantibacillus plantarum* 299v	Probiotic bacteria	Isolate from commercial product SanProbi IBS
*Lactobacillus helveticus*	Probiotic bacteria	ATCC 27558
*Lacticaseibacillus rhamnosus* GG	Probiotic bacteria	ATCC 53103
*Bacillus subtilis*	Spore-forming bacteria causing food spoilage	Isolate from food
*Enterococcus faecalis*	Antibiotic-resistant faecal bacteria; an indicator of faecal contamination	Isolate from food
*Listeria monocytogenes*	Pathogenic bacteria: parasites of animals and humans	ATCC 19111
*Staphylococcus aureus*	Pathogenic bacteria with enterotoxigenic properties	ATCC 25923
Gram-negative bacteria		
*Escherichia coli*	Intestinal bacteria; sanitary indicator	ATCC 10536
*Pseudomonas aeruginosa*	Pathogenic bacteria resistant to antibiotics	ATCC 15442
*Salmonella enterica*	Pathogenic bacteria causing food poisoning	Clinical isolate from WSSE

**Table 2:** Analysis of the contents of bioactive compounds in the obtained haskap berry extracts.

	TPC * [mg/g DL **]	Loganic Acid [mg/g DL **]	Chlorogenic Acid [mg/g DL **]	Caffeic Acid [mg/g DL **]	Rutin [mg/g DL **]	Quercetin [mg/g DL **]
Extract 1	54.127 ± 1.214 ^a^	2.814 ± 0.155 ^b^	0.989 ± 0.068 ^a^	0.080 ± 0.002 ^a^	0.895 ± 0.117 ^a^	1.314 ± 0.115 ^a^
Extract 2	56.256 ± 1.304 ^b^	2.877 ± 0.151 ^b^	1.024 ± 0.077 ^a^	0.082 ± 0.003 ^a^	0.912 ± 0.122 ^a,b^	1.349 ± 0.110 ^a^
Extract 3	60.014 ± 1.311 ^c^	2.982 ± 0.164 ^b^	1.127 ± 0.089 ^b^	0.097 ± 0.005 ^b^	1.157 ± 0.131 ^c^	1.509 ± 0.127 ^c^
Extract 4	58.259 ± 1.287 ^b^	2.824 ± 0.152 ^b^	1.007 ± 0.074 ^a^	0.089 ± 0.005 ^b^	0.949 ± 0.120 ^b^	1.407 ± 0.111 ^b^
Extract 5	51.057 ± 1.109 ^a^	2.754 ± 0.137 ^a^	0.956 ± 0.061 ^a^	0.077 ± 0.004 ^a^	0.871 ± 0.114 ^a^	1.249 ± 0.099 ^a^

* TPC—total polyphenol content; ** DL—dry weight of leaves. Data expressed as mean ± SD; columns with different superscript letters (^a–c^) differ significantly (*p* < 0.05).

**Table 3:** Analysis of the content of bioactive compounds in the prepared dextran systems.

	TPC * [mg/g DL **]	Loganic Acid [mg/g DL **]	Chlorogenic Acid [mg/g DL **]	Caffeic Acid [mg/g DL **]	Rutin [mg/g DL **]	Quercetin [mg/g DL **]
System 1 (Dextran 5.000)	59.124 ± 1.214	2.907 ± 0.161	1.124 ± 0.087	0.095 ± 0.004	1.129 ± 0.124	1.497 ± 0.124
System 2 (Dextran 40.000)	59.667 ± 1.127	2.980 ± 0.155	1.125 ± 0.084	0.093 ± 0.005	1.143 ± 0.122	1.507 ± 0.127
System 3 (Dextran 70.000)	59.551 ± 1.124	2.974 ± 0.153	1.125 ± 0.083	0.092 ± 0.003	1.137 ± 0.121	1.501 ± 0.121
Extract 3 (reference)	60.014 ± 1.311	2.982 ± 0.164	1.127 ± 0.089	0.097 ± 0.005	1.157 ± 0.131	1.509 ± 0.127

* TPC—total polyphenol content; ** DL—dry weight of leaves. No statistically significant differences (*p* < 0.05) were found between the samples and the base extract.

**Table 4:** Analysis of the antioxidant potential and biological activity of obtained dextran systems.

	System 1	System 2	System 3	Extract 3 (Reference)
Antioxidant potential expressed as Trolox equivalent [mg/mL]				
ABTS	0.2312 ± 0.0095	0.2514 ± 0.0103	0.2447 ± 0.0101	0.2412 ± 0.0109
CUPRAC	0.3056 ±0.0113	0.3215 ±0.0119	0.3121 ± 0.0115	0.3156 ± 0.0099
DPPH	0.2005 ±0.0063	0.2245 ±0.0070	0.21001 ± 0.0066	0.2106 ± 0.0084
FRAP	0.3288 ± 0.0124	0.3647 ± 0.0137	0.3411 ± 0.0129	0.3488 ± 0.0147
Biological activity				
α-glucosidase expressed as acarbose equivalent [mg/mL]	1.5048 ± 0.0571	1.5541 ± 0.05889	1.4915 ± 0.0565	1.5848 ± 0.0659
Hyaluronidase inhibition expressed as quercetin equivalent [mg/mL]	0.3052 ± 0.0122	0.3144 ± 0.0126	0.2982 ± 0.0119	0.3245 ± 0.0129
Lipase inhibition expressed as Orlistat equivalent [µg/mL]	5.9781 ± 0.2397	6.1112 ± 0.2397	5.8715 ± 0.2397	6.1271 ± 0.2433

No statistically significant differences (*p* < 0.05) were found between the samples and the base extract.

**Table 5:** The microbial analysis over 72 h of different bacterial strains across the control and dextran systems. Means with (*) within the same column within the same time point differ significantly from control (*p* < 0.05).

	Time [H]	Control	System 1	System 2	System 3	Dextran 5000	Dextran 40,000	Dextran 70,000
*Bifidobacterium longum*	24	1.08 × 10^9^	2.61 × 10^8^ *	2.73 × 10^8^ *	2.05 × 10^8^ *	8.29 × 10^7^ *	9.41 × 10^7^ *	9.54 × 10^7^ *
	48	1.30 × 10^9^	1.49 × 10^9^	1.49 × 10^9^	1.82 × 10^9^	1.40 × 10^9^	1.23 × 10^9^	1.57 × 10^10^ *
	72	1.09 × 10^9^	1.34 × 10^9^	1.58 × 10^9^	1.00 × 10^9^	1.27 × 10^9^	9.59 × 10^8^ *	1.35 × 10^10^ *
*Bifidobacterium animalis*	24	1.62 × 10^8^	2.61 × 10^8^	3.17 × 10^8^ *	2.75 × 10^8^	1.72 × 10^8^	1.21 × 10^8^	8.53 × 10^7^ *
	48	3.21 × 10^8^	5.65 × 10^8^ *	4.25 × 10^8^	4.71 × 10^8^ *	2.60 × 10^8^	2.96 × 10^8^	2.91 × 10^8^
	72	2.05 × 10^8^	5.78 × 10^8^ *	1.56 × 10^9^ *	7.32 × 10^8^ *	2.65 × 10^8^	2.70 × 10^8^	3.41 × 10^7^ *
*Faecalibacterium prausnitzii*	24	4.82 × 10^8^	1.32 × 10^9^ *	9.64 × 10^8^	1.12 × 10^9^ *	5.49 × 10^8^	5.14 × 10^8^	4.71 × 10^8^
	48	7.45 × 10^8^	1.55 × 10^9^ *	1.08 × 10^9^ *	1.12 × 10^9^ *	4.71 × 10^8^	4.23 × 10^8^	3.48 × 10^8^
	72	2.27 × 10^8^	8.62 × 10^8^ *	8.67 × 10^8^ *	5.99 × 10^8^	1.34 × 10^8^	1.87 × 10^8^	2.01 × 10^8^
*Lactobacillus salivarius*	24	1.48 × 10^10^	1.54 × 10^10^	1.98 × 10^10^	2.03 × 10^10^	1.54 × 10^10^	1.47 × 10^10^	1.36 × 10^10^
	48	1.36 × 10^10^	1.42 × 10^10^	1.37 × 10^10^	1.38 × 10^10^	1.27 × 10^10^	1.53 × 10^10^	1.12 × 10^9^ *
	72	1.72 × 10^10^	3.31 × 10^10^	2.84 × 10^10^	2.86 × 10^10^	1.75 × 10^10^	1.69 × 10^10^	9.75 × 10^8^ *
*Lactiplantibacillus plantarum* 299v	24	1.13 × 10^10^	1.98 × 10^10^	1.52 × 10^10^	1.65 × 10^10^	1.25 × 10^10^	1.10 × 10^10^	1.42 × 10^10^
	48	1.33 × 10^10^	1.18 × 10^10^	1.25 × 10^10^	2.13 × 10^10^	1.32 × 10^10^	1.32 × 10^10^	1.24 × 10^10^
	72	1.73 × 10^10^	3.41 × 10^10^	2.99 × 10^10^	3.03 × 10^10^ *	1.73 × 10^10^	1.47 × 10^10^	1.62 × 10^10^
*Lactobacillus helveticus*	24	4.17 × 10^8^	6.20 × 10^8^	8.82 × 10^8^	9.59 × 10^8^	4.46 × 10^8^	3.39 × 10^8^	2.96 × 10^8^
	48	4.00 × 10^8^	8.02 × 10^8^ *	6.68 × 10^8^	6.95 × 10^8^	3.42 × 10^8^	3.81 × 10^8^	4.30 × 10^8^
	72	3.81 × 10^8^	1.16 × 10^9^ *	8.67 × 10^8^	1.29 × 10^9^ *	3.06 × 10^8^	3.15 × 10^8^	4.15 × 10^8^
*Lacticaseibacillus rhamnosus* GG	24	2.28 × 10^9^	1.57 × 10^10^ *	1.96 × 10^9^	1.61 × 10^9^	2.16 × 10^9^	1.77 × 10^9^	3.48 × 10^8^ *
	48	5.74 × 10^9^	2.22 × 10^10^ *	9.53 × 10^9^	1.22 × 10^10^ *	7.32 × 10^9^	6.24 × 10^9^	1.59 × 10^9^
	72	2.23 × 10^10^	4.15 × 10^10^	3.44 × 10^10^	2.79 × 10^10^	1.90 × 10^10^	2.13 × 10^10^	1.32 × 10^10^

## Data Availability

The data are contained within the article or Supplementary Material.

## Supplementary Materials

The following supporting information can be downloaded at: https://www.mdpi.com/article/10.3390/antiox13030357/s1, Table S1—The microbial analysis over 72 h of different potentially pathogenic bacterial strains across the control and dextran systems. No statistically significant differences were found (p < 0.05). Table S2—HPLC method validation parameters. Figure S1—Standard curve for the conversion of TPC expressed as gallic acid equivalent. Figure S2—standard curve for the conversion of antioxidant activity in the ABTS assay for Trolox. Figure S3—standard curve for the conversion of antioxidant activity in the CUPRAC assay for Trolox. Figure S4—standard curve for the conversion of antioxidant activity in the DPPH assay for Trolox. Figure S5—standard curve for the conversion of antioxidant activity in the FRAP assay for Trolox.
