# Supplementary material for: Exploring Beneficial Properties of Haskap Berry Leaf Compounds for Gut Health Enhancement
Source: Antioxidants (Basel). 2024 Mar 17;13(3):357. doi: 10.3390/antiox13030357 (PMC10968585; doi:10.3390/antiox13030357)
Supplement: Supplementary file 1 [file antioxidants-13-00357-s001.zip › antioxidants-2812423-supplementary.pdf]

antioxidants

Article

Exploring Beneficial Properties of Haskap Berry Leaf Compounds for Gut Health Enhancement

Szymon Sip [1], Anna Sip [2], Piotr Szulc [3], Marek Selwet [4], Marcin Żarowski [5], Bogusław Czerny [6] and Judyta Cielecka-Piontek [1,7,*]

1 Department of Pharmacognosy and Biomaterials, Faculty of Pharmacy, Poznań University of Medical Sciences, Rokietnicka 3, 60-806 Poznań, Poland; szymonsip@ump.edu.pl
2 Department of Biotechnology and Food Microbiology, Poznan University of Life Sciences, Wojska Polskiego 48, 60-627 Poznań, Poland; anna.sip@up.poznan.pl
3 Department of Agronomy, Poznań University of Life Sciences, Dojazd 11, 60-632 Poznań, Poland; piotr.szulc@up.poznan.pl
4 Department of Soil Science and Microbiology, Poznań University of Life Sciences, Szydłowska 50, 60-656 Poznań, Poland; marek.selwet@up.poznan.pl
5 Department of Developmental Neurology, Poznan University of Medical Sciences, Przybyszewski 49, 60-355 Poznan, Poland; zarowski@ump.edu.pl
6 Department of General Pharmacology and Pharmacoeconomics, Pomeranian Medical University in Szczecin, Żołnierska 48, 70-204 Szczecin, Poland; bczerny@wp.pl
7 Department of Pharmacology and Phytochemistry, Institute of Natural Fibres and Medicinal Plants, Wojska Polskiego 71b, 60-630 Poznan, Poland
* Correspondence: jpiontek@ump.edu.pl

Abstract: This study investigates the potential of formulated systems utilising haskap berry leaf extracts and dextran as carriers, to modulate both antioxidant and enzymatic inhibitory activities and their impact on the growth of specific bacterial strains. The analysis of antioxidant capacity, assessed through ABTS, CUPRAC, DPPH, and FRAP assays, revealed varying but consistently high levels across extracts, with Extract 3 (loganic acid: 2.974 mg/g, chlorogenic acid: 1.125 mg/g, caffeic acid: 0.083 mg/g, rutin: 1.137 mg/g, and quercetin: 1.501 mg/g) exhibiting the highest values (ABTS: 0.2447 mg/mL, CUPRAC: 0.3121 mg/mL, DPPH: 0.21001 mg/mL, and FRAP: 0.3411 mg/mL). Subsequent enzymatic inhibition assays demonstrated a notable inhibitory potential against α-glucosidase (1.4915 mg/mL, expressed as acarbose equivalent), hyaluronidase (0.2982 mg/mL, expressed as quercetin equivalent), and lipase (5.8715 µg/mL, expressed as orlistat equivalent). Further system development involved integration with dextran, showcasing their preserved bioactive compound content and emphasising their stability and potential bioactivity. Evaluation of the dextran systems' impact on bacterial growth revealed a significant proliferation of beneficial strains, particularly the *Bifidobacterium* and lactobacilli genus (Bifidobacterium longum: 9.54×10^7 to 1.57×10^{10} CFU/mL and *Ligilactobacillus salivarius*: 1.36×10^9 to 1.62×10^{10} CFU/mL), suggesting their potential to modulate gut microbiota. These findings offer a foundation for exploring the therapeutic applications of haskap berry-based dextran systems in managing conditions like diabetes, emphasising the interconnected roles of antioxidant-rich botanical extracts and dextran formulations in promoting overall metabolic health.

Keywords: haskap berry; prebiotic potential; antioxidants; by-products; delivery systems; dextran

Table S1. The microbial analysis over 72 hours of different potentially pathogenic bacterial strains across the control and dextran systems. No statistically significant differences were found ($p < 0.05$)

	Time [H]	Control	System 1	System 2	System 3	Dextran 5000	Dextran 40 000	Dextran 70 000
Bacillus subtilis	24	7.82×10^8	8.52×10^8	8.88×10^8	8.21×10^8	7.29×10^8	7.53×10^8	7.65×10^8
	48	1.48×10^9	1.29×10^9	1.31×10^9	1.40×10^9	1.36×10^9	1.33×10^9	1.41×10^9
	72	1.13×10^9	1.11×10^9	1.19×10^9	1.03×10^9	1.16×10^9	1.08×10^9	1.22×10^9
Enterococcus faecalis	24	1.45×10^8	1.81×10^8	2.03×10^8	1.92×10^8	1.71×10^8	1.62×10^8	1.55×10^8
	48	3.17×10^8	3.32×10^8	3.22×10^8	3.38×10^8	3.27×10^8	3.29×10^8	3.32×10^8
	72	2.22×10^8	2.12×10^8	2.38×10^8	2.19×10^8	2.34×10^8	2.28×10^8	2.09×10^8
Listeria monocytogenes	24	4.39×10^8	4.51×10^8	4.31×10^8	4.41×10^8	4.61×10^8	4.51×10^8	4.41×10^8
	48	7.15×10^8	7.31×10^8	7.21×10^8	7.31×10^8	7.41×10^8	7.31×10^8	7.21×10^8
	72	2.09×10^8	2.32×10^8	2.42×10^8	2.21×10^8	2.26×10^8	2.21×10^8	2.11×10^8
Staphylococcus aureus	24	1.37×10^9	1.51×10^9	1.62×10^9	1.73×10^9	1.52×10^9	1.43×10^9	1.34×10^9
	48	1.18×10^9	1.42×10^9	1.31×10^9	1.42×10^9	1.37×10^9	1.39×10^9	1.24×10^9
	72	1.72×10^9	1.81×10^9	1.71×10^9	1.81×10^9	1.76×10^9	1.71×10^9	1.61×10^9
Escherichia coli	24	1.12×10^9	1.21×10^9	1.11×10^9	1.16×10^9	1.11×10^9	1.06×10^9	1.13×10^9
	48	1.26×10^9	1.31×10^9	1.26×10^9	1.36×10^9	1.31×10^9	1.33×10^9	1.30×10^9
	72	1.59×10^9	1.79×10^9	1.69×10^9	1.79×10^9	1.74×10^9	1.69×10^9	1.59×10^9
Pseudomonas aeruginosa	24	3.83×10^8	4.30×10^8	4.00×10^8	4.20×10^8	4.10×10^8	4.00×10^8	3.90×10^8
	48	3.89×10^8	4.10×10^8	3.90×10^8	4.00×10^8	3.90×10^8	3.80×10^8	3.90×10^8
	72	3.79×10^8	3.90×10^8	3.80×10^8	3.90×10^8	3.80×10^8	3.70×10^8	3.90×10^8
Salmonella enterica	24	2.29×10^8	2.30×10^8	2.20×10^8	2.30×10^8	2.10×10^8	2.00×10^8	2.20×10^8
	48	5.56×10^8	5.80×10^8	5.60×10^8	5.80×10^8	5.70×10^8	5.50×10^8	5.70×10^8
	72	2.08×10^8	2.30×10^8	2.20×10^8	2.30×10^8	2.20×10^8	2.10×10^8	2.20×10^8

Table S2. HPLC method validation parameters

Parameter	Loganic acid	Chlorogenic acid	Caffeic acid	Rutin	Quercetin
Linearity: y = ax + b	y= 126,74x + 0,2999	y = 564.25x + 2.6133	y = 1298.4x - 0.3044	y = 1164.9x - 0.2995	y = 1455.3x - 0.3184
Correlation coefficient (*r*)	0,9999	0,9998	0,9998	0,9997	0,9998
Range of linearity [mg/ml]	0,01-5,00	0,01-5,00	0,001-1,00	0,01-5,00	0,01-5,00
Limit of detection (LOD): [mg/ml]	1,2590 x 10^{-4}	2,1125 x 10^{-4}	1,1249 x 10^{-4}	2,3114 x 10^{-4}	1,6530 x 10^{-4}
Limit of quantification (LOQ): [mg/ml]	4,1547x 10^{-4}	6,9713 x 10^{-4}	3,7121 x 10^{-4}	7,6276 x 10^{-4}	5,4549 x 10^{-4}
Intra-day precision, RSD					
0,001 mg/ml	1,08 %	1,19 %	1,14 %	1,14 %	1,11 %
0,05 mg/ml	1,13 %	1,11 %	1,15 %	1,33 %	1,22 %
0,1 mg/ml	1,11%	1,24 %	1,15 %	1,13 %	1,12 %
Inter-day precision, RSD					
0,001 mg/ml	1,14 %	1,39 %	1,09 %	1,04 %	1,19 %
0,05 mg/ml	1,20 %	1,21%	1,09 %	1,05 %	1,16 %
0,1 mg/ml	1,35 %	1,25 %	1,03 %	1,13 %	1,15 %
Accuracy	99,59 %	99,54 %	99,68 %	99,69 %	99,65 %

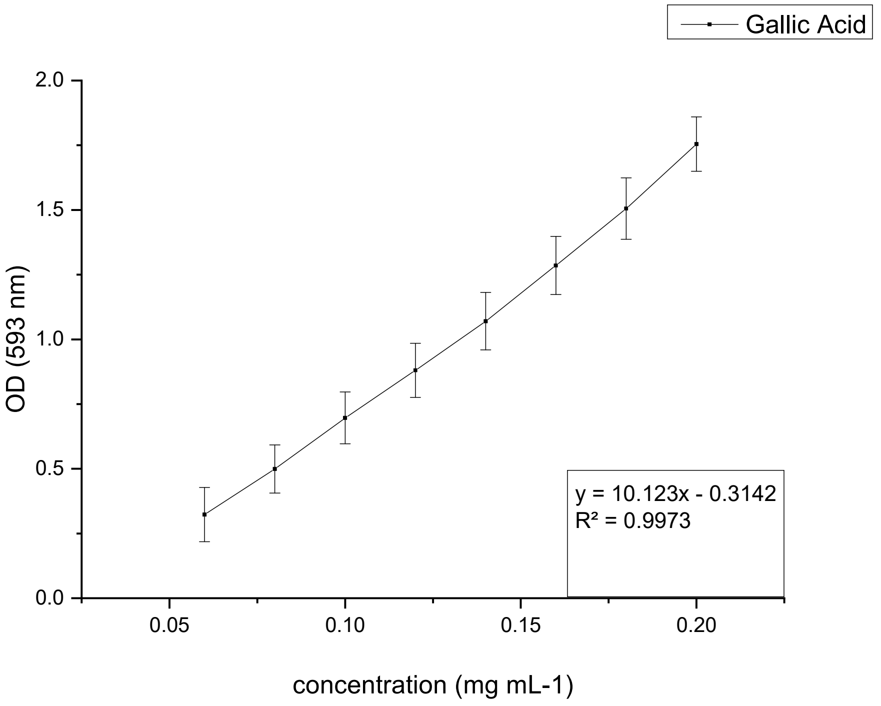

Figure S1. Standard curve for the conversion of TPC expressed as gallic acid equivalent

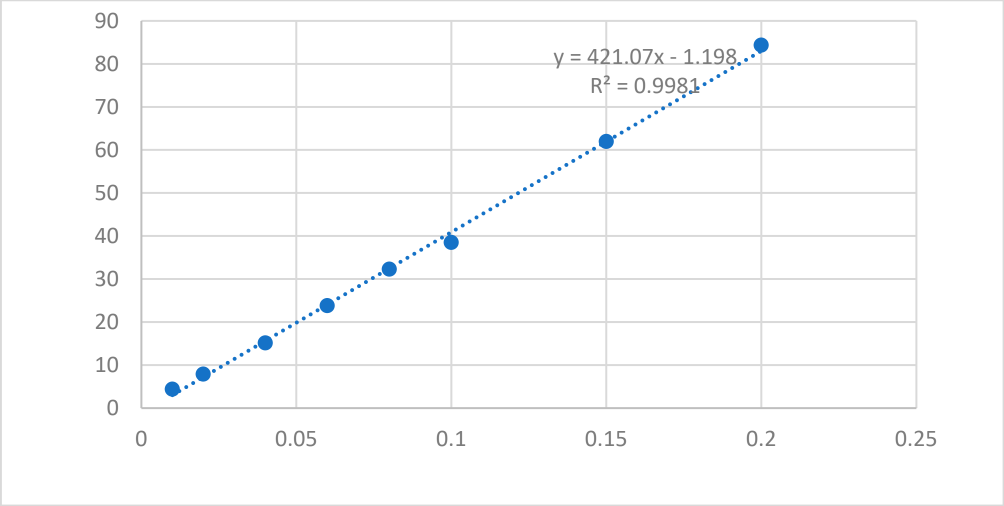

Figure S2. Standard curve for the conversion of antioxidant activity in the ABTS assay for Trolox

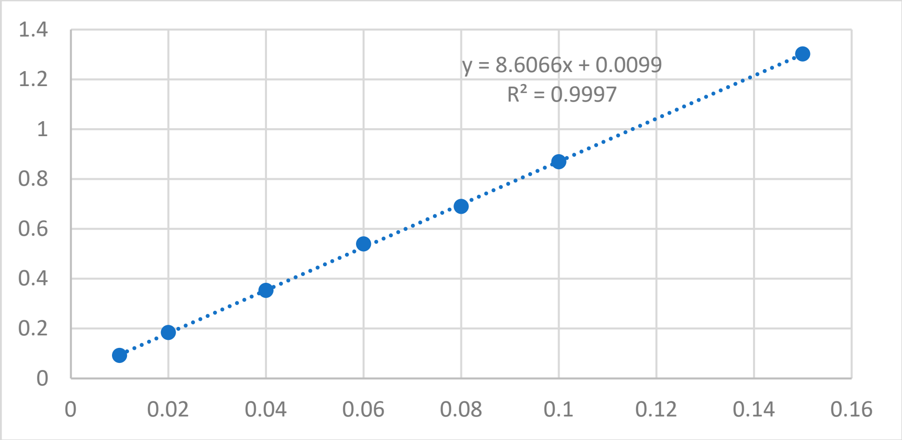

Figure S3. Standard curve for the conversion of antioxidant activity in the CUPRAC assay for Trolox

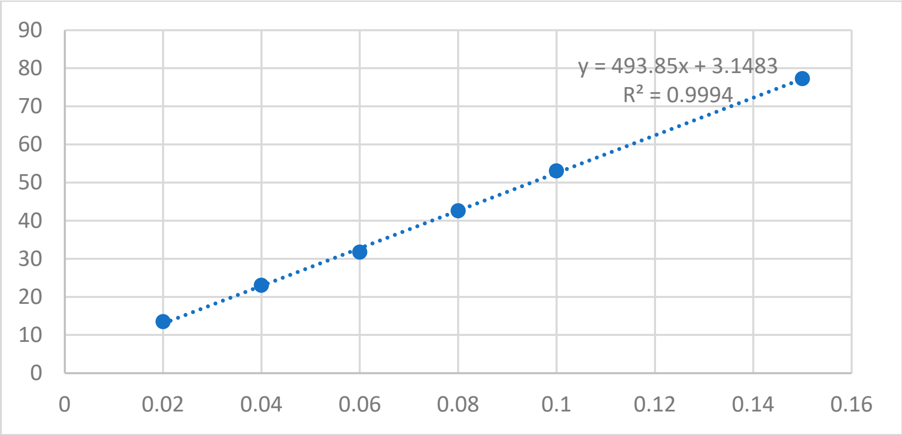

Figure S4. Standard curve for the conversion of antioxidant activity in the DPPH assay for Trolox

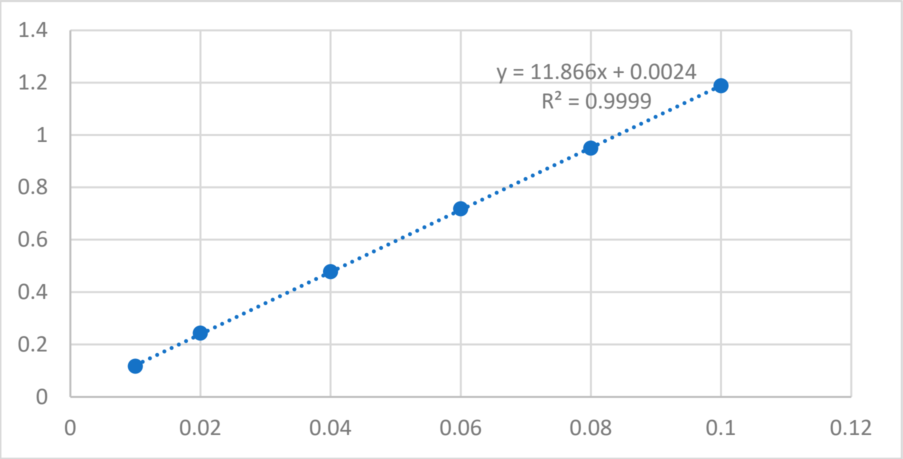

Figure S5. Standard curve for the conversion of antioxidant activity in the FRAP assay for Trolox
